# Immunogenetic profiles of 9 human herpes virus envelope glycoproteins

**DOI:** 10.1038/s41598-024-71558-1

**Published:** 2024-09-09

**Authors:** Apostolos P. Georgopoulos, Lisa M. James

**Affiliations:** 1 The HLA Research Group, Brain Sciences Center, Department of Veterans Affairs Health Care System, Minneapolis VAMC, One Veterans Drive, Minneapolis, MN 55417 USA; 2 Department of Neuroscience, University of Minnesota Medical School, Minneapolis, MN USA; 3 Institute for Health Informatics, University of Minnesota Medical School, Minneapolis, MN USA; 4 Department of Psychiatry, University of Minnesota Medical School, Minneapolis, MN USA

**Keywords:** Human herpes viruses, Binding affinity, Human leukocyte antigen (HLA), Computational biology and bioinformatics, Genetics, Immunology, Microbiology, Diseases, Medical research

## Abstract

Human herpes viruses (HHV) are ubiquitous and have been implicated in numerous long-term health conditions. Since the association between viral exposure and long-term health impacts is partially influenced by variation in human leukocyte antigen (HLA) genes, we evaluated in silico the binding affinities of 9 HHV envelope glycoproteins with 127 common HLA Class I and Class II molecules. The findings show substantial variability in HHV binding affinity across viruses, HLA Class, HLA genes, and HLA alleles. Specific findings were as follows: (1) the predicted binding affinities of HHVs were characterized by four distinct groupings—[HHV1, HHV2], [HHV3, HHV4, HHV5], [HHV6A], [HHV6B, HHV7, HHV8]—with relatively lower binding affinities for HHV1, HHV2, and HHV6a compared to other HHVs; (2) significantly higher binding affinity was found for HLA Class I relative to Class II; (3) analyses within each class demonstrated that alleles of the C gene (for Class I) and DRB1 gene (for Class II) had the highest binding affinities; and (4) for each virus, predicted binding affinity to specific alleles varied, with HHV6a having the lowest affinity for HHV-HLA complexes, and HHV3, HHV4, and HHV5 having the highest. Since HLA-antigen binding is the first step in initiating an immune response to foreign antigens, these relative differences in HHV binding affinities are likely to influence long-term health impacts such that the cells infected with viruses associated with higher binding affinities across common HLA alleles may be more reduced in numbers, thereby lowering the potential for long-term sequelae of their infections.

## Introduction

Human herpes viruses (HHV) are practically ubiquitous viruses that can establish lifelong infection characterized by alternating periods of latency and reactivation^1–3^. HHVs include herpes simplex virus 1 (HSV1/HHV1) and HSV2/HV2, varicella zoster virus (VZV/HHV3), Epstein-Barr virus (EBV/ HHV4), cytomegalovirus (CMV/HHV5), HHV6A, HHV6B, HHV7, and Kaposi’s sarcoma virus (HHV8). As a family of viruses, herpesviridae tropism spans multiple systems^4^, and HHVs have been implicated in numerous long-term health conditions including autoimmune disorders, neoplasms, and neurodegenerative conditions^5,6^.

The association between viral exposure and long-term health impacts is partially influenced by individual variation in human leukocyte antigen (HLA) genes^7–10^. Located on chromosome 6, the HLA region is the most highly polymorphic of the human genome^11^. Small differences, even single amino acid changes in the binding groove, can alter HLA-antigen binding^12^, thereby influencing foreign antigen elimination and disease susceptibility^8,11^. HLA genes code for cell-surface glycoproteins that present bound viral epitopes to T cells, signaling immune system responses aimed at virus elimination. Each individual possesses 12 HLA alleles, inherited in a Mendelian fashion, including two of each of the HLA Class I genes (HLA-A, HLA-B, and HLA-C) and two of each of the HLA Class II genes (HLA-DR, HLA-DQ, HLA-DP). Glycoproteins of the two classes operate in concert albeit via different mechanisms and timeframes. HLA Class I, which are expressed on all nucleated cells, signal destruction of an infected cell by binding and transporting cytosolic virus epitopes to the cell surface for presentation to cytotoxic CD8 + T cells. HLA Class II molecules, which are expressed on lymphocytes and professional antigen presenting cells, bind and present endocytosed exogenous antigen epitopes to CD4 + T cells to stimulate antibody production and long-term adaptive immunity. Developing long-term immunity in the event of virus re-exposure occurs over weeks to months compared to the rapid elimination of infected cells via the HLA Class I system^13^.

It is reasonable to hypothesize that rapid elimination of viral antigens by HLA Class I molecules may reduce viral latency and, consequently, reduce long-term sequelae of viral infections. In light of the high degree of HLA polymorphism, however, the immune response to a HLA-virus antigen complex varies, meaning that some HLA-antigen complexes will be more efficient in mounting an immune response to a given virus than others. Indeed, this is exemplified by slowed progression and control of human immunodeficiency virus (HIV) in carriers of certain HLA alleles (for example, HLA-B*27:05 and B*57:01), as compared to rapid disease progression associated with other HLA alleles (e.g., B*35:01)^14^. A recent review synthesizing genetic associations of HLA with herpesvirus infection and disease found that most HLA genetic associations are virus- or disease- specific, although certain allotypes were broadly associated with susceptibility or control across herpes viruses^15^. We hypothesize that observed differences in HLA associations with HHVs are related to immune response of HHV-HLA pairs, the first step of which hinges on sufficient HLA-virus antigen binding affinity. Thus, in this study we used an in silico approach to evaluate the binding affinity of 9 HHV proteins with 127 common HLA Class I (n = 69) and Class II (n = 58) antigens.

## Results

### Predicted binding affinities (PBA) of HHV proteins with HLA class I and II molecules

**Fig. 1:**
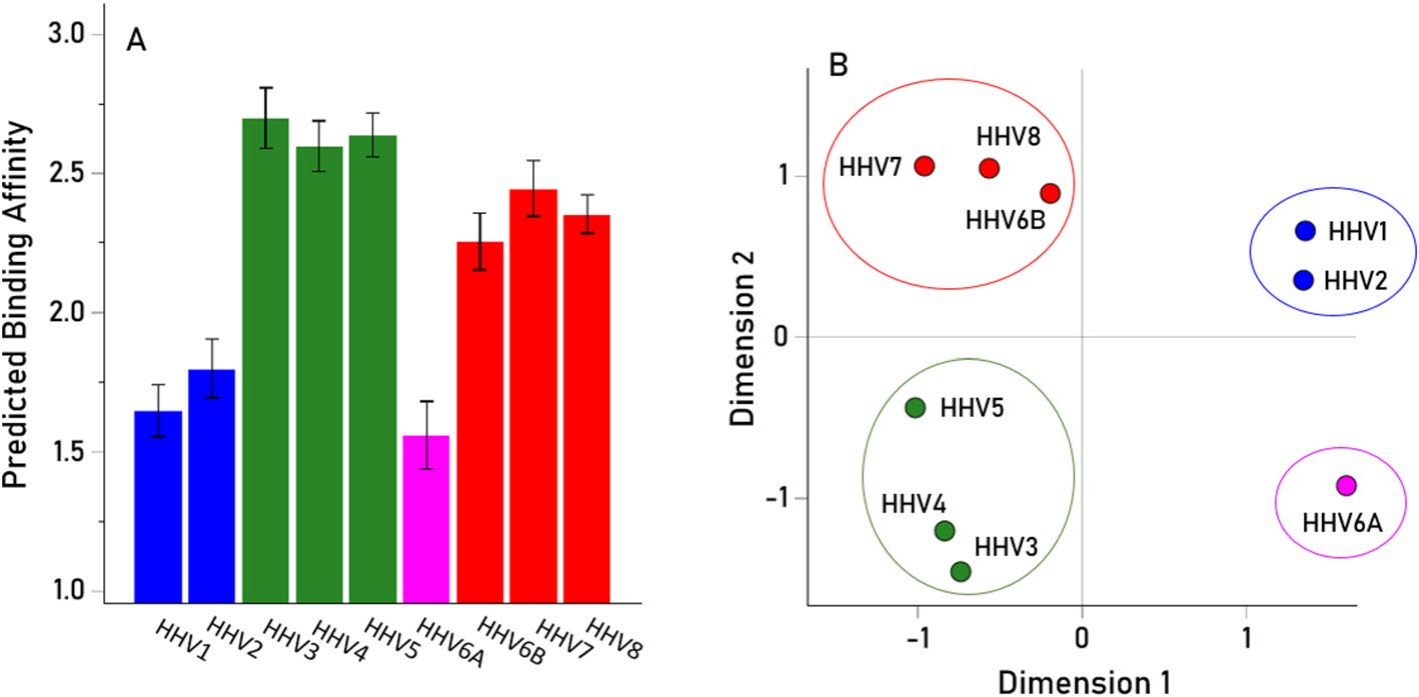
(**A**) Mean predicted binding affinity (± SEM) of the 9 HHV proteins analyzed across the 127 HLA alleles. (**B**) Plot of the derived HHV protein configuration yielded by the multidimensional scaling analysis. Viral proteins are color-coded to highlight the 4 distinct PBA groups.

The HHV PBAs of the 9 HHV (Table 1) and 127 HLA Class I and II alleles (Tables 2 and 3) are given in Table S1 in Supplementary Material. The effects of HLA Class on these PBAs were evaluated using a repeated measures analysis of variance (ANOVA), where HHV PBA was the “Within-Subjects” factor and HLA Class was the “Between-Subjects” fixed factor. The results revealed a highly significant effect of HHV on PBA (Fig. 1A; P < 0.001, Greenhouse–Geisser test) and four distinct PBA groupings (color-coded in Fig. 1A): [HHV1, HHV2], [HHV3, HHV4, HHV5], [HHV6A], [HHV6B, HHV7, HHV8]. The same groupings were found using multidimensional scaling (MDS), occupying 4 distinct quadrants in the MDS map (Fig. 1B).

**Table 1:** Viral proteins used.

Index	Virus	Protein description	UniprotKB ID	AA
1	HHV1	Envelope glycoprotein D	Q69091	394
2	HHV2	Envelope glycoprotein D	P03172	393
3	HHV3	Envelope glycoprotein E	Q9J3M8	623
4	HHV4	Envelope glycoprotein B	P03188	897
5	HHV5	Envelope glycoprotein B	P06473	906
6	HHV6A	Envelope glycoprotein Q2	P0DOE0	214
7	HHV6B	Envelope glycoprotein Q1	Q9QJ11	516
8	HHV7	Envelope glycoprotein H	P52353	690
9	HHV8	Envelope glycoprotein H	F5HAK9	730

**Table 2:** The 69 HLA Class I alleles used.

Index	Gene A	Index	Gene B	Index	Gene C
1	A*01:01	21	B*07:02	57	C*01:02
2	A*02:01	22	B*08:01	58	C*03:03
3	A*02:05	23	B*13:02	59	C*04:01
4	A*03:01	24	B*14:01	60	C*05:01
5	A*11:01	25	B*14:02	61	C*06:02
6	A*23:01	26	B*15:01	62	C*07:01
7	A*24:02	27	B*15:17	63	C*07:02
8	A*25:01	28	B*15:18	64	C*07:04
9	A*26:01	29	B*18:01	65	C*12:02
10	A*29:01	30	B*27:02	66	C*12:03
11	A*29:02	31	B*27:05	67	C*14:02
12	A*30:01	32	B*35:01	68	C*15:02
13	A*30:02	33	B*35:02	69	C*16:01
14	A*31:01	34	B*35:03		
15	A*32:01	35	B*35:08		
16	A*33:01	36	B*37:01		
17	A*33:03	37	B*38:01		
18	A*36:01	38	B*39:01		
19	A*68:01	39	B*39:06		
20	A*68:02	40	B*40:01		
		41	B*40:02		
		42	B*41:01		
		43	B*41:02		
		44	B*44:02		
		45	B*44:03		
		46	B*44:05		
		47	B*45:01		
		48	B*47:01		
		49	B*49:01		
		50	B*50:01		
		51	B*51:01		
		52	B*52:01		
		53	B*55:01		
		54	B*56:01		
		55	B*57:01		
		56	B*58:01

**Table 3:** The 58 HLA Class II alleles used.

Index	Gene	Index	Gene	Index	Gene
1	DPB1*01:01	16	DQB1*02:01	30	DRB1*01:01
2	DPB1*02:01	17	DQB1*02:02	31	DRB1*01:02
3	DPB1*02:02	18	DQB1*03:01	32	DRB1*01:03
4	DPB1*03:01	19	DQB1*03:02	33	DRB1*03:01
5	DPB1*04:01	20	DQB1*03:03	34	DRB1*04:01
6	DPB1*04:02	21	DQB1*04:02	35	DRB1*04:02
7	DPB1*05:01	22	DQB1*05:01	36	DRB1*04:03
8	DPB1*06:01	23	DQB1*05:02	37	DRB1*04:04
9	DPB1*09:01	24	DQB1*05:03	38	DRB1*04:05
10	DPB1*10:01	25	DQB1*06:01	39	DRB1*04:07
11	DPB1*11:01	26	DQB1*06:02	40	DRB1*04:08
12	DPB1*13:01	27	DQB1*06:03	41	DRB1*07:01
13	DPB1*14:01	28	DQB1*06:04	42	DRB1*08:01
14	DPB1*17:01	29	DQB1*06:09	43	DRB1*08:03
15	DPB1*19:01			44	DRB1*09:01
				45	DRB1*10:01
				46	DRB1*11:01
				47	DRB1*11:02
				48	DRB1*11:03
				49	DRB1*11:04
				50	DRB1*12:01
				51	DRB1*13:01
				52	DRB1*13:02
				53	DRB1*13:03
				54	DRB1*13:05
				55	DRB1*14:01
				56	DRB1*15:01
				57	DRB1*15:02
				58	DRB1*16:01

**Fig. 2:**
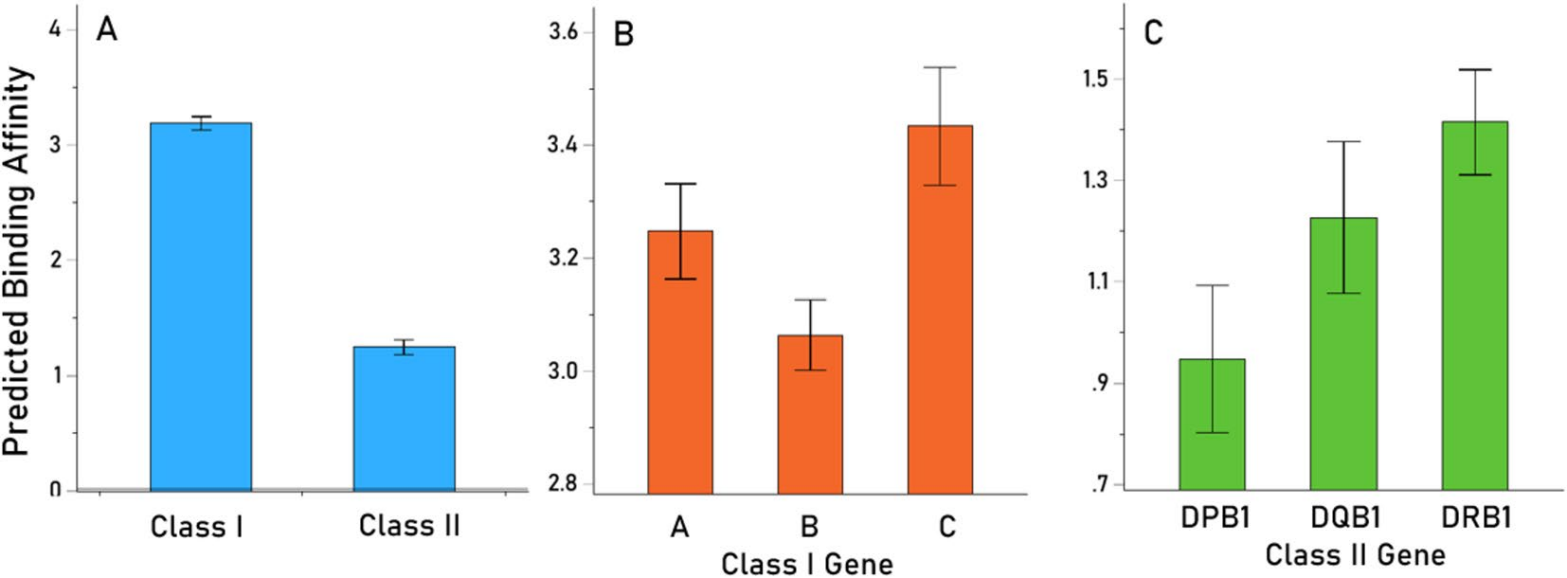
**A** Mean predicted binding affinity (± SEM) of the 9 HHV proteins in the 2 HLA Classes (**A**), Class I genes (**B**), and Class II genes (**C**).

With respect to HLA Class, Class I PBA was significantly higher than Class II (Fig. 2A, P < 0.001, F-test). Finally, with respect to HLA genes, we used a repeated measures ANOVA to evaluate the effect of Gene (“Between-Subjects fixed factor) within each Class. We found a statistically significant effect of Gene for both Class I (P = 0.01, F-test) and Class II (P = 0.038, F-test), with genes C and DRB1 having the highest PBAs (Fig. 2B,C, respectively).

### Variation of PBA across HLA alleles

**Fig. 3:**
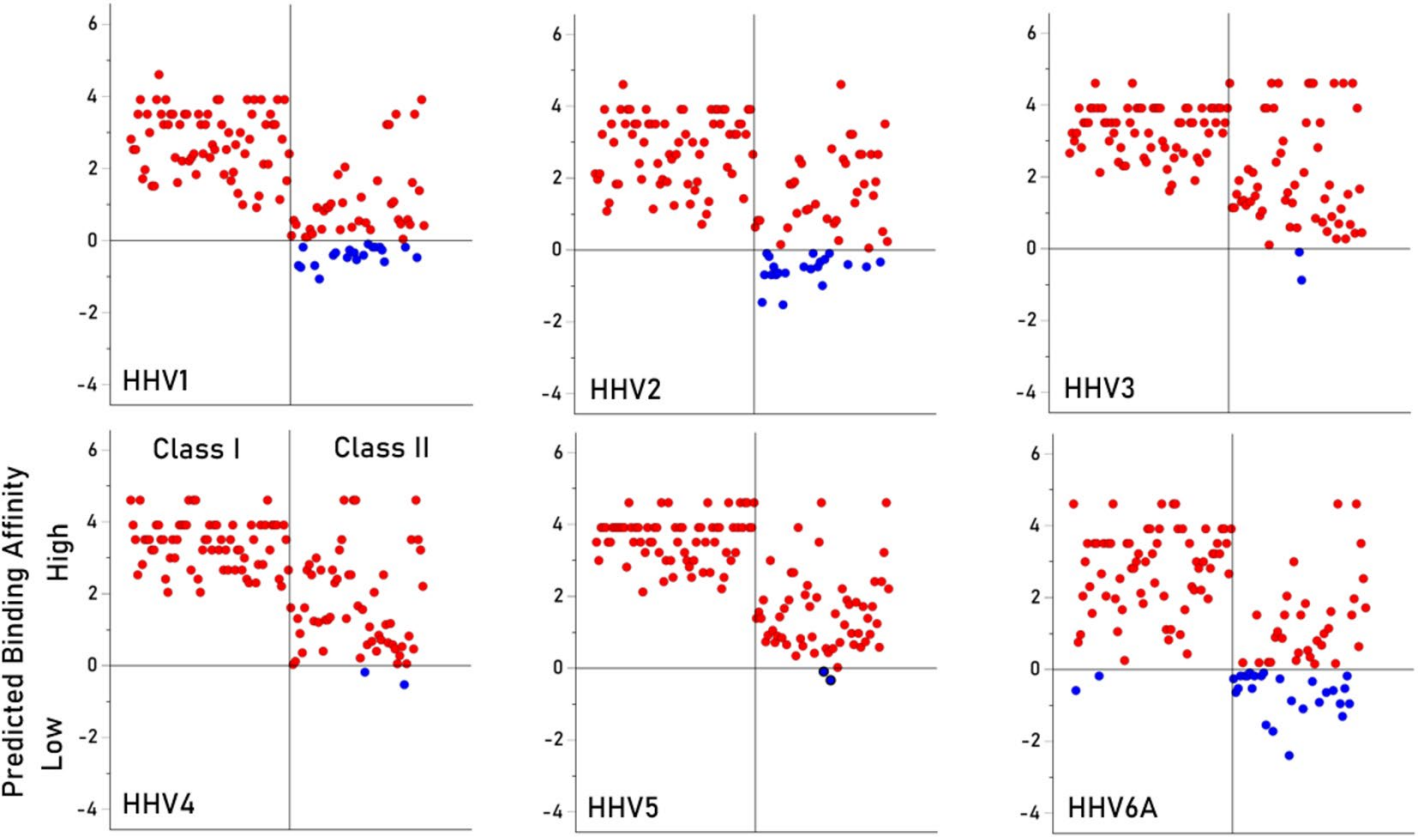
Individual PBA values are plotted for viruses HHV1–HHV6A, as indicated. Red, high affinity PBA; blue, low affinity PBA. N = 127 for each plot.

**Fig. 4:**
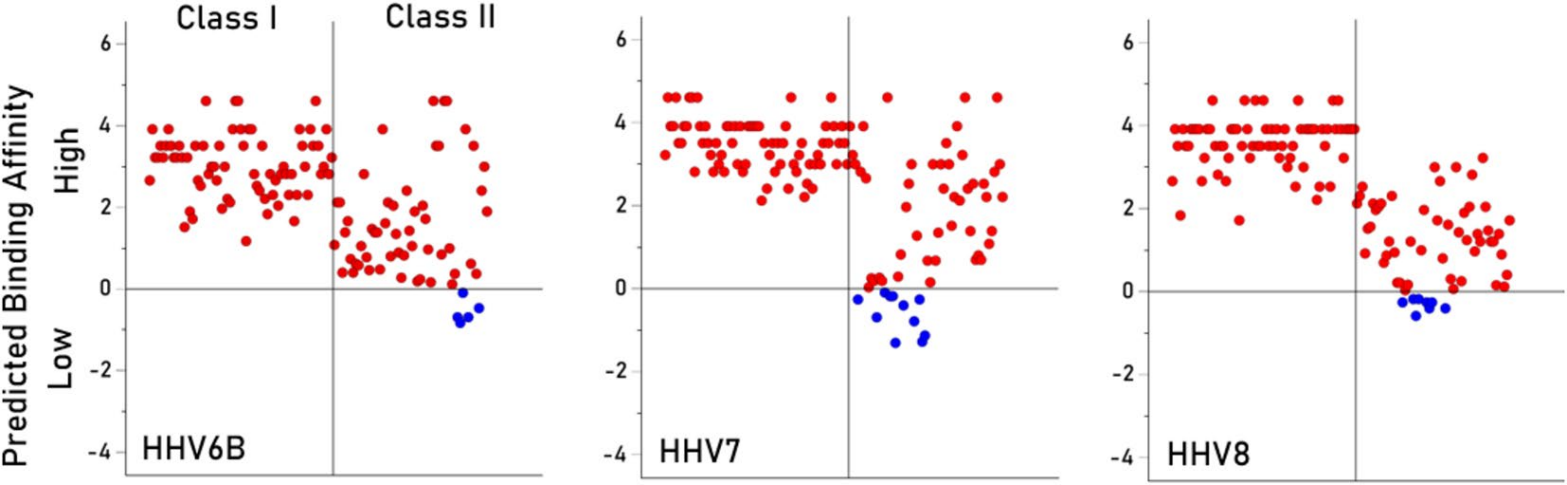
Individual PBA values are plotted for viruses HHV6B, HHV7 and HHV8, as indicated. Red, high affinity PBA; blue, low affinity PBA. N = 127 for each plot.

**Fig. 5:**
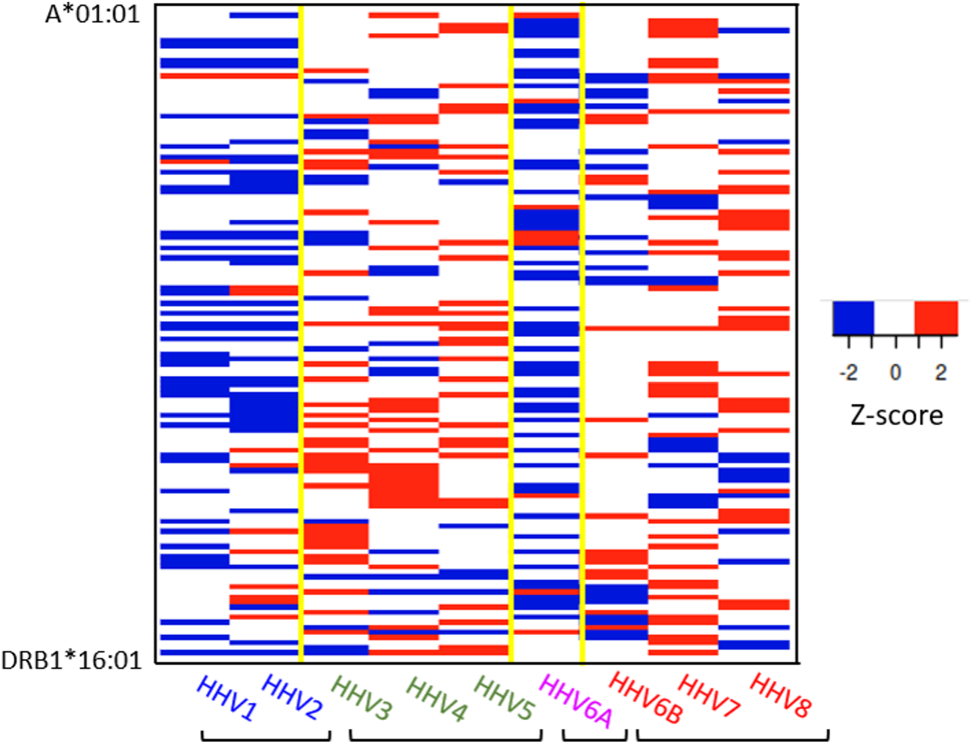
Heatmap of PBAs across the 127 HLA alleles (rows) and the 9 viral proteins (columns). Red, high PBA values (Z-score ≥ 2); blue, low PBA values (Z-score ≤ -2).

The analyses above evaluated the overall effects of Virus, Class and Gene on PBA. Here we show the individual PBA values for all 127 HLA alleles and the 9 HHV viruses in Figs. 3 and 4. The PBA value of zero corresponds to lowest percentile rank of 1 (PBA = ln(1) = 0; see Methods), a conservative threshold for high binding affinity. It can be seen that (a) most PBAs are of high affinity (> 0), (b) the number of low affinity PBA differ across viruses, being highest for HHV6A and lowest for HHV3, HHV4 and HHV5, and (c) in all viruses but HHV6A, low affinity PBAs are confined to Class II. This variation in PBA across alleles and viruses is captured in the heatmap of Fig. 5.

### Association of PBA with protein length

**Fig. 6:**
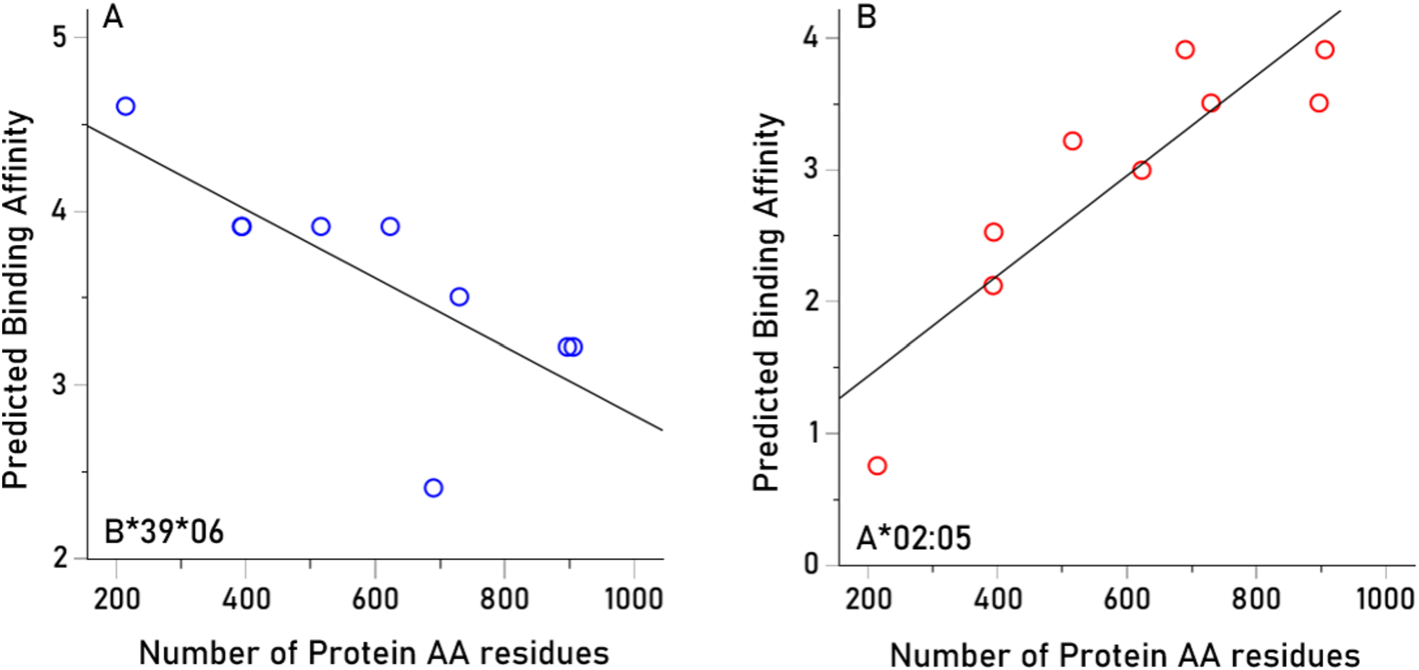
(**A**) negative association between PBA and number of amino acid (AA) residues of the HHV proteins (Table 1) for allele B*39:06. (**B**) positive association, for allele A*02:05.

Since we tested all possible 9-AA (for Class I) and 15-AA (for Class II) epitopes, it is possible that PBA estimates could depend on protein length since longer proteins would afford more AA sequences to which HLA molecules could potentially bind. We evaluated this hypothesis by computing, for each allele, the Pearson correlation between PBA values and the number of amino acids in a protein (Table 1). We found the following (Table 4). (a) 18/127 (14.1%) of the correlations were negative (10 Class I, 8 Class II), speaking against the hypothesis above; an example is shown in Fig. 6A. (b) 95/127 (74.8%) correlations were not statistically significant, at a nominal threshold of P < 0.05, uncorrected for multiple comparisons; no correlation was significant at a threshold of P < 0.05/127, i.e. P < 0.000394, after the conservative Bonferroni correction or P < 0.000404, after the less conservative Šidák correction (see Methods); and (c) the percent of PBA variance explained by protein AA length (i.e. 100 x ${r}^{2}$), irrespective of statistical significance, ranged from 0.001 to 78.5%, but was heavily skewed towards small values (median = 16.7%, mean ± SEM 24.9 ± 2.1%). An example of high positive correlation is shown in Fig. 6B. Overall, these results indicate that there is only a small overall contribution of protein length to PBA values.

**Table 4:** Correlation between HHV PBA and the number of amino acids in the HHV protein (N = 9). *, P < 0.05.

Index	HLA allele	Class	Gene	$r$ Pearson	P-value	Percent of variance explained
1	A*01:01	1	1	0.1414	0.7167	1.999
2	A*02:01	1	1	0.7334	0.0245*	53.793
3	A*02:05	1	1	0.8864	0.0015*	78.568
4	A*03:01	1	1	0.3691	0.3284	13.620
5	A*11:01	1	1	0.7155	0.0302*	51.197
6	A*23:01	1	1	0.5215	0.1499	27.193
7	A*24:02	1	1	0.4754	0.1958	22.605
8	A*25:01	1	1	0.6672	0.0496*	44.516
9	A*26:01	1	1	0.8258	0.0061*	68.197
10	A*29:01	1	1	0.5064	0.1642	25.647
11	A*29:02	1	1	0.5064	0.1642	25.647
12	A*30:01	1	1	0.5019	0.1686	25.190
13	A*30:02	1	1	0.1697	0.6625	2.879
14	A*31:01	1	1	− 0.0005	0.9989	0.000
15	A*32:01	1	1	0.7196	0.0288*	51.786
16	A*33:01	1	1	− 0.0346	0.9296	0.120
17	A*33:03	1	1	− 0.1377	0.7239	1.896
18	A*36:01	1	1	− 0.5571	0.1192	31.038
19	A*68:01	1	1	0.6239	0.0726	38.920
20	A*68:02	1	1	0.8610	0.0029*	74.129
21	B*07:02	1	2	0.3514	0.3538	12.347
22	B*08:01	1	2	0.4557	0.2177	20.766
23	B*13:02	1	2	0.7441	0.0215*	55.363
24	B*14:01	1	2	0.2255	0.5596	5.085
25	B*14:02	1	2	0.2255	0.5596	5.085
26	B*15:01	1	2	0.6041	0.0849	36.497
27	B*15:17	1	2	0.3886	0.3013	15.102
28	B*15:18	1	2	0.5747	0.1055	33.030
29	B*18:01	1	2	0.8092	0.0082*	65.481
30	B*27:02	1	2	0.1412	0.7171	1.993
31	B*27:05	1	2	0.1222	0.7541	1.494
32	B*35:01	1	2	0.6533	0.0564	42.684
33	B*35:02	1	2	− 0.1272	0.7443	1.619
34	B*35:03	1	2	− 0.2384	0.5368	5.682
35	B*35:08	1	2	0.6203	0.0747	38.483
36	B*37:01	1	2	0.6997	0.0359*	48.952
37	B*38:01	1	2	0.3403	0.3701	11.584
38	B*39:01	1	2	− 0.1490	0.7019	2.221
39	B*39:06	1	2	− 0.7481	0.0204*	55.959
40	B*40:01	1	2	0.5616	0.1156	31.535
41	B*40:02	1	2	0.7892	0.0114*	62.286
42	B*41:01	1	2	0.7848	0.0123*	61.584
43	B*41:02	1	2	0.4623	0.2102	21.375
44	B*44:02	1	2	0.1335	0.7320	1.783
45	B*44:03	1	2	0.2514	0.5140	6.322
46	B*44:05	1	2	0.1068	0.7846	1.140
47	B*45:01	1	2	0.8818	0.0017*	77.754
48	B*47:01	1	2	0.2151	0.5784	4.627
49	B*49:01	1	2	0.7972	0.0101*	63.560
50	B*50:01	1	2	0.7929	0.0108*	62.868
51	B*51:01	1	2	− 0.2813	0.4634	7.913
52	B*52:01	1	2	0.2988	0.4348	8.926
53	B*55:01	1	2	0.4628	0.2096	21.422
54	B*56:01	1	2	0.1705	0.6610	2.907
55	B*57:01	1	2	− 0.0663	0.8654	0.440
56	B*58:01	1	2	0.1480	0.7040	2.190
57	C*01:02	1	3	0.0073	0.9850	0.005
58	C*03:03	1	3	0.3188	0.4030	10.164
59	C*04:01	1	3	0.6436	0.0614	41.419
60	C*05:01	1	3	0.7428	0.0219*	55.175
61	C*06:02	1	3	0.1482	0.7036	2.196
62	C*07:01	1	3	0.8779	0.0019*	77.075
63	C*07:02	1	3	0.6494	0.0584	42.169
64	C*07:04	1	3	0.7295	0.0257*	53.212
65	C*12:02	1	3	0.3373	0.3747	11.377
66	C*12:03	1	3	0.0723	0.8533	0.523
67	C*14:02	1	3	0.2842	0.4585	8.079
68	C*15:02	1	3	0.5929	0.0924	35.158
69	C*16:01	1	3	0.2565	0.5052	6.581
70	DPB1*01:01	2	4	0.4997	0.1708	24.969
71	DPB1*02:01	2	4	0.4088	0.2746	16.714
72	DPB1*02:02	2	4	0.4069	0.2771	16.554
73	DPB1*03:01	2	4	0.7532	0.0191*	56.736
74	DPB1*04:01	2	4	0.5051	0.1655	25.509
75	DPB1*04:02	2	4	0.4019	0.2836	16.153
76	DPB1*05:01	2	4	0.8815	0.0017*	77.703
77	DPB1*06:01	2	4	0.7597	0.0175*	57.714
78	DPB1*09:01	2	4	0.7099	0.0322*	50.392
79	DPB1*10:01	2	4	0.7479	0.0205*	55.933
80	DPB1*11:01	2	4	0.2491	0.5181	6.205
81	DPB1*13:01	2	4	0.4097	0.2735	16.784
82	DPB1*14:01	2	4	0.7369	0.0235	54.305
83	DPB1*17:01	2	4	0.6677	0.0494*	44.579
84	DPB1*19:01	2	4	0.4835	0.1873	23.380
85	DQB1*02:01	2	5	0.2349	0.5430	5.517
86	DQB1*02:02	2	5	0.2349	0.5430	5.517
87	DQB1*03:01	2	5	0.1720	0.6581	2.959
88	DQB1*03:02	2	5	0.3846	0.3068	14.790
89	DQB1*03:03	2	5	0.0720	0.8539	0.519
90	DQB1*04:02	2	5	0.0819	0.8341	0.670
91	DQB1*05:01	2	5	0.4846	0.1862	23.483
92	DQB1*05:02	2	5	0.3653	0.3337	13.343
93	DQB1*05:03	2	5	0.2830	0.4607	8.006
94	DQB1*06:01	2	5	0.7545	0.0188*	56.927
95	DQB1*06:02	2	5	0.7915	0.0110*	62.651
96	DQB1*06:03	2	5	− 0.0974	0.8031	0.950
97	DQB1*06:04	2	5	0.5744	0.1057	32.993
98	DQB1*06:09	2	5	0.6394	0.0637	40.879
99	DRB1*01:01	2	6	0.1778	0.6471	3.163
100	DRB1*01:02	2	6	0.2987	0.4349	8.923
101	DRB1*01:03	2	6	0.1621	0.6770	2.626
102	DRB1*03:01	2	6	− 0.1666	0.6684	2.775
103	DRB1*04:01	2	6	− 0.1313	0.7363	1.724
104	DRB1*04:02	2	6	0.4284	0.2499	18.356
105	DRB1*04:03	2	6	0.1163	0.7657	1.353
106	DRB1*04:04	2	6	0.3001	0.4327	9.006
107	DRB1*04:05	2	6	0.0633	0.8714	0.401
108	DRB1*04:07	2	6	0.0113	0.9771	0.013
109	DRB1*04:08	2	6	0.0170	0.9653	0.029
110	DRB1*07:01	2	6	0.7908	0.0112*	62.534
111	DRB1*08:01	2	6	− 0.4362	0.2405	19.025
112	DRB1*08:03	2	6	− 0.4704	0.2013	22.130
113	DRB1*09:01	2	6	0.5371	0.1359	28.848
114	DRB1*10:01	2	6	0.1782	0.6465	3.175
115	DRB1*11:01	2	6	− 0.6965	0.0371*	48.514
116	DRB1*11:02	2	6	0.2902	0.4488	8.421
117	DRB1*11:03	2	6	0.3671	0.3311	13.475
118	DRB1*11:04	2	6	0.7600	0.0175*	57.761
119	DRB1*12:01	2	6	0.0863	0.8253	0.744
120	DRB1*13:01	2	6	0.2902	0.4488	8.421
121	DRB1*13:02	2	6	0.2990	0.4345	8.939
122	DRB1*13:03	2	6	− 0.0736	0.8508	0.541
123	DRB1*13:05	2	6	− 0.6965	0.0371*	48.514
124	DRB1*14:01	2	6	0.7490	0.0202*	56.100
125	DRB1*15:01	2	6	0.2329	0.5465	5.424
126	DRB1*15:02	2	6	0.0310	0.9369	0.096
127	DRB1*16:01	2	6	0.5425	0.1313	29.432

## Discussion

Here we evaluated binding affinities of 127 common HLA Class I and Class II alleles with envelope glycoproteins of 9 HHVs and documented substantial variability in the predicted binding affinities of HHVs with regard to HLA Class, gene, and allele. Since HLA–HHV antigen binding is a critical initial step in mounting an adaptive immune response to a viral infection, these findings highlight relative differences in binding affinities of specific HHVs with common HLA Class I and Class II alleles and point to enhanced ability of certain HLA alleles to facilitate a more effective adaptive immune system response to HHVs that bind with higher affinity to common HLA alleles. In the absence of high affinity HLA-HHV complex binding, the virus may persist^16^, establish latency^17^, and contribute to subsequent long-term health impacts^18,19^.

The HLA-HHV binding affinities here fell into four distinct groups, two of which were characterized by distinctly lower binding affinities. Specifically, HHV1/HHV2 and HHV6a, neurotropic viruses that have been implicated (to varying degrees) with neurological conditions^20–23^, had low binding affinities overall (Fig. 1) and particularly for HLA Class II (Figs. 3, 4, 5). HHV1 and HHV2, commonly referred to as herpes simplex virus -1 (HSV-1) and -2 (HSV-2), cause lifelong infections characterized by periods of latency and reactivation in the form of orolabial (HSV-1) or genital (HSV-2) lesions. The seroprevalence of HSV-1, which is typically acquired by oral contact, in individuals between the ages of 14 and 49 is estimated at 54% whereas the seroprevalence of HSV-2, which is typically sexually transmitted, is 16% in the same age range^24^. Extensive research supports a prominent role of HSV-1 in dementia^20^; HSV-2 has been shown to increase HIV acquisition^25^ and is associated with neurological complications^26^. Part of the roseola family, HHV6A infection is very common early in life, and, as a neurotropic virus, is commonly detected in the brains of healthy individuals as well as those with neurological diseases^27–29^. Notably, HHV6A has been shown to integrate into the host germline, allowing for generational transmission of the HHV6A viral genome^30^. The relatively low binding affinity of HHV1, HHV2, and HHV6A with common HLA alleles may hinder efficient elimination of those viruses, potentially contributing to the development of subsequent neurological effects. Despite their relatively higher overall binding affinities, several of the HHVs comprising the other two groupings (HHV3, HHV4, HHV5; HHV6b, HHV7, HHV8) have also been implicated in long-term health conditions including various cancers, neurological, and autoimmune disorders^19,31–35^. It is worth noting that even for the 6 viruses associated with higher overall predicted binding affinities to common HLA alleles, there was still substantial variability in predicted binding affinity across alleles (Figs. 3, 4, 5), indicating that some are preferentially able to bind with high affinity compared to others. Thus, the findings suggest that some HHVs are overall more readily handled by HLA-mediated adaptive immune system mechanisms than others, due to their superior binding affinity with common HLA alleles. Nonetheless, for all of the HHVs, the effectiveness of the adaptive immune system response is predicated on high-affinity HLA-HHV binding. To that end, we hypothesize that HHV latency and reactivation as well as long-term disease associations are HLA-dependent.

It is noteworthy that the predicted binding affinities of HHVs with Class I HLA molecules were significantly higher than those of Class II. HLA Class I and Class II play different albeit complementary roles in the adaptive immune response to viruses. HLA Class I promotes rapid elimination of infected cells via cytotoxic CD8 + T cells, whereas Class II contributes to long-term protection via antibody production and immunological memory, a process that can take months^13^. We propose that rapid elimination of HHVs by binding with HLA Class I to form high affinity HHV-HLA complexes may reduce the potential of the virus to establish latency, thereby reducing the likelihood of viral reactivation and associated diseases, as has been established in the case of early efficient elimination of HIV by certain Class I alleles^36^. This rapid elimination via Class I mediated cytotoxic T cells does not preclude development of Class II mediated antibody production; indeed, much of the population is seropositive for one or more HHVs (1,2,3). In some cases, seropositivity is associated with disease^19,37^, suggesting that antibodies reflective of seropositivity do not necessarily confer protection. For both HLA Class I and II, however, adaptive immune protection against HHVs are HLA-dependent in that absence of sufficient HHV-HLA binding affinity inhibits presentation to CD8 + or CD4 + T cells necessary for signaling destruction of infected cells (Class I) or antibody production (Class II), permitting the viral antigen to persist.

Class I HLA-C and Class II HLA-DRB1 genes were associated with higher binding affinity to HHVs than other genes within each respective HLA class. Compared to other classical HLA Class I genes (A and B), HLA-C is unique in that it is less frequently expressed on the cell surface but is the only HLA Class I gene for which virtually all allotypes serve as a natural ligand for multiple types of killer-cell immunoglobulin-like receptors (KIR) which are expressed on natural killer cells that are known to control infected cells efficiently^38^. As reviewed elsewhere^38^, mounting research has documented that HLA-C, in combination with KIR, influences control and/or progression of various viral infections including HIV, hepatitis C, and CMV, a member of the herpesviridae family (HHV5). Alleles of the DRB1 gene have been associated with both protection and susceptibility to various conditions including numerous autoimmune disorders^7,39,40^, many of which are associated with virus exposure^41^. The current study shines the spotlight more prominently on Class I HLA-C and Class II HLA-DRB1 in influencing the outcome and progression of various HHVs and points to superior binding affinity of HLA-C and HLA-DRB1 as an important underlying mechanism.

The present findings, which document that binding affinity of a given HHV varies across HLA Class I and Class II alleles, must be considered with several qualifications. First, to ensure their survival, HHVs are notorious for utilizing immune evasion mechanisms, several of which involve downregulation of HLA or interference with transport or loading of antigenic peptides which may impair viral elimination even in the case of a strong antiviral immune response^42^. Second, the focus here is on virus antigen–HLA binding since that is a necessary first step in adaptive immunity; the extent of the human immune response is also partially dependent on immunogenicity of the antigen-HLA complex. Thus, it is possible that some of the high affinity virus-HLA associations documented here may not produce a sufficient immunogenic response against viral antigens. Third, the analyses focused on 127 common HLA Class I and Class II alleles; it is possible that other less common alleles that were not investigated here are capable of forming highly immunogenic complexes. Nonetheless, focusing on globally common alleles permits greater generalization of the findings. Finally, for each virus, we analyzed binding affinity of a single protein of a single strain—specifically, an envelope glycoprotein that is involved in viral entry into the cell. It is unclear to what extent the present findings extend to other proteins and other strains of each of the viruses investigated; although such analyses are beyond the scope of the present paper, they are currently underway.

## Materials and methods

### HHV proteins

We estimated the binding affinity (for each one of the 69 Class I alleles and 58 Class II alleles) of envelope glycoproteins of 9 HHV viruses (HHV1, HHV2, HHV3, HHV4, HHV5, HHV6A, HHV6B, HHV7, HHV8). Details of the proteins analyzed are given in Table 1 and their amino acid (AA) sequences are given in Table 2S in Supplementary Material. These proteins are involved in virus entry into the cell^43^ have been widely used in HHV-immunology research, including vaccine development^44–53^.

### HLA alleles

We used 69 common HLA Class I alleles (Table 2) and 58 common HLA Class II alleles (Table 3) that we have employed in previous studies^54^. Briefly, we obtained the population frequency in 2019 of 127 common HLA Class I and Class II alleles from 14 Continental Western European Countries (Austria, Belgium, Denmark, Finland, France, Germany, Greece, Italy, Netherlands, Portugal, Norway, Spain, Sweden, and Switzerland). There was a total of 2746 entries of alleles from these countries, comprising 844 distinct alleles. Of those, 69 Class I alleles and 58 Class II alleles occurred in 9 or more countries, with a minimum frequency (in any country) of 0.01. Although those alleles were selected based on their frequency in Europe, they have been found to the common overall across 6 world populations^55^, namely African/African American (AFA), Asian/Pacific Islands (API), European/European descent (EURO), Middle East/North Coast of Africa (MENA), South or Central America/Hispanic/Latino (HIS), Native American (NAM), Unknown/not asked/multiple ancestries/other (UKN), and total (TOTAL). All but allele A*36:01 were Common in each one of the 6 populations above; allele A*36:01 was Intermediate in API and EURO populations, and Common in the remainder populations, and was Common across the 6 populations.

### In silico* determination of predicted binding affinity of HLA Class I and Class II molecules*

**Fig. 7:**
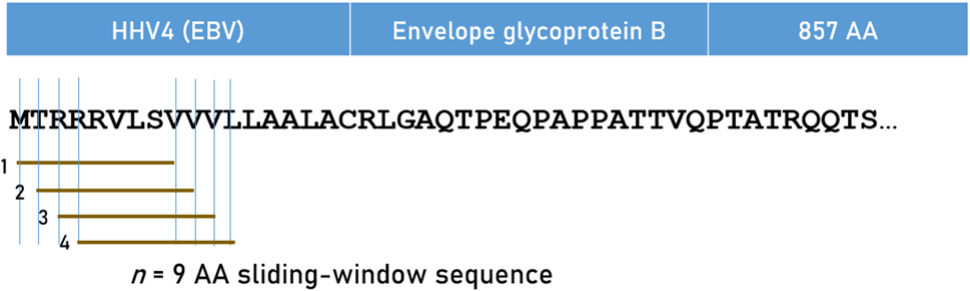
Sliding window method for HLA Class I analyses (window length = 9 AA), illustrated for HHV4.

**Fig. 8:**
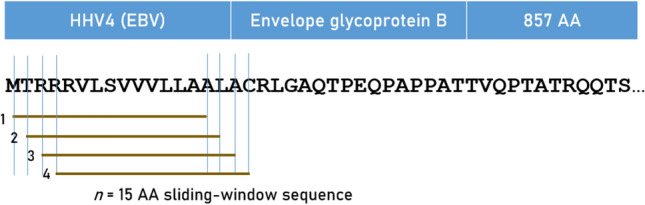
Sliding window method for HLA Class II analyses (window length = 15 AA), illustrated for HHV4.

Predicted binding affinities were obtained for viral protein epitopes using the Immune Epitope Database (IEDB) NetMHCpan (ver. 4.1) tool^56,57^. More specifically, we used the sliding window approach^58–60^ to test exhaustively all possible linear 9-mer (for HLA-I predictions) and 15-mer (for HLA-II predictions) AA residue epitopes of the 9 viral proteins analyzed (Table 1). The method is illustrated in Figs. 7 and 8 for the HHV4 virus protein. For each epitope-HLA molecule tested, this tool gives, as an output, the percentile rank of binding affinity of the HLA molecule and the epitope among predicted binding affinities of the same HLA molecule to a large number of different peptides of the same AA length; the smaller the percentile rank, the better the binding affinity. Now, given a protein of *N* amino acid length and an epitope length of *k* AA, there are *N-k* binding affinity predictions, i.e. *N-k* percentile ranks. Of these predictions, for each viral protein and HLA molecule tested, we retained the lowest percentile rank (LPR) as the best possible binding affinity of the protein-HLA molecule pair. We then applied two transformations on LPR. First, we took its inverse, so that higher values mean better binding affinities for more intuitive interpretation:

1 $$LP{R}^{\prime}=\frac{1}{LPR}$$

**Fig. 9:**
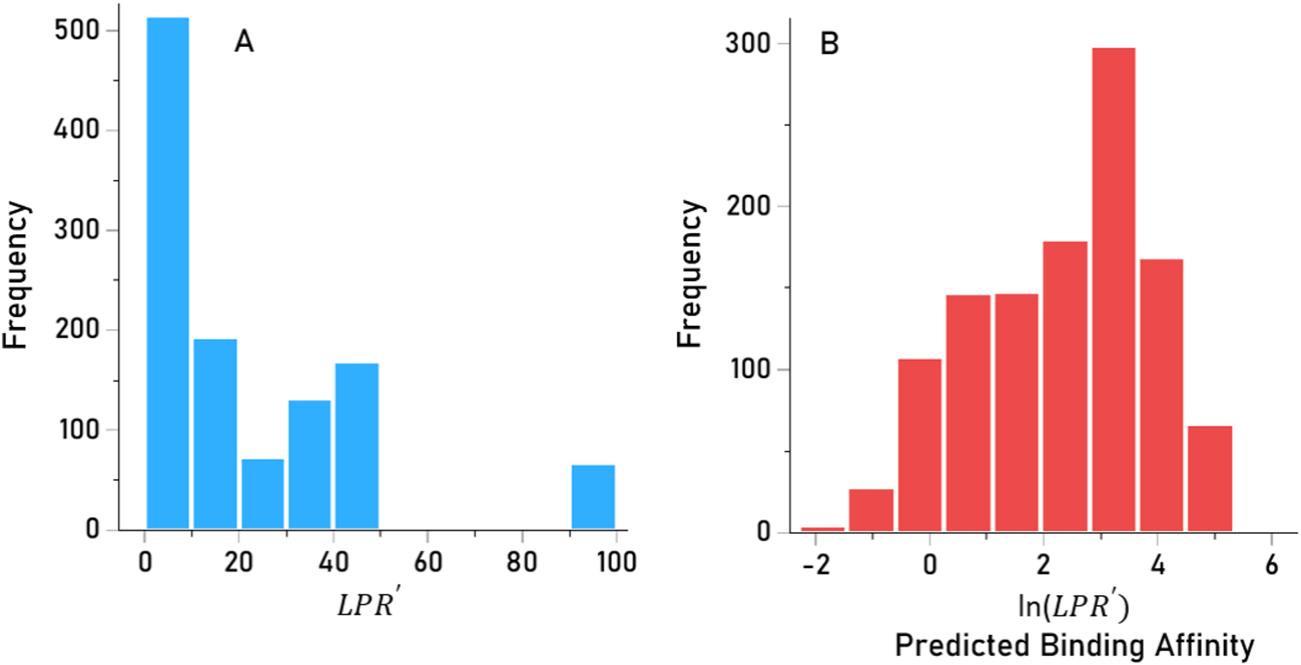
Frequency distributions of $LPR^{\prime}$ (Eq. 1) (**A**, skewed to the left) and its log-transformed PBA values (Eq. 2) (**B**, unimodal).

The $LPR^{\prime}$ distribution was heavily skewed to the left (Fig. 9A), resembling an exponential distribution. Therefore, $LPR^{\prime}$ values were (natural) log transformed to normalize its distribution for quantitative analyses (Fig. 9B):

2 $$\text{Predicted Binding Affinity }(\text{PBA}): PBA = \text{ln}(LP{R}^{\prime})$$

Give the logarithmic transformation above, PBA > 0 indicate $LP{R}^{\prime}>1$, whereas PBA < 0 indicate $LP{R}^{\prime}<1$.

### Statistical analyses

The IBM-SPSS statistical package (version 29.0.1.1 244) was used for implementing statistical analyses. Standard statistical methods were used, including descriptive statistics, ANOVA, Pearson correlation, etc. All P-values reported are 2-sided, $a=0.05$.

### Statistical significance uncorrected for multiple comparisons

For this condition, with $a=0.05$, P < 0.05 indicated a statistically significant effect for each one of 127 correlations computed between viral PBA and number of amino acids in a viral protein.

### Statistical significance corrected for multiple comparisons

Here we computed P values adjusted for the 127 correlations above in two ways, as follows. The first adjustment was the Bonferroni correction, where

3 $$\text{P}(\text{Bonferroni adjusted}) =\frac{0.05}{127}=0.000394$$

The second was the Šidák correction, where

4 $$\text{P}({\mathop{\text{S}}\limits^v}\text{id}\acute{a}\text{k adjusted}) =1-{\left(1-0.05\right)}^\frac{1}{127}=1-0.999596=0.000404$$

### Multidimensional scaling

The potential groupings of the 9 HHV PBAs were evaluated by MDS using the ALSCAL procedure with the following parameters: Model: Euclidean distance; Level of measurement: ratio; Conditionality: matrix; Dimensions: minimum 2, maximum 2; S-stress convergence: 0.0001, minimum s-stress value: 0.005, maximum number of iterations: 30).

### Visualization

For PBA visualization, the Heatmapper tool^61^ was used (http://www.heatmapper.ca/; accessed on July 13, 2024).

## Supplementary Information

Supplementary Information.

## Data Availability

All data used were retrieved from freely accessible websites and, as such, are publicly and freely available at http://www.biostatistics.online/ineo-epp/neoantigen.php].
