# Supplementary material for: Immunogenetic profiles of 9 human herpes virus envelope glycoproteins
Source: Sci Rep. 2024 Sep 9;14:20924. doi: 10.1038/s41598-024-71558-1 (PMC11385983; doi:10.1038/s41598-024-71558-1)
Supplement: Supplementary file 1 — Supplementary Information. [file 41598_2024_71558_MOESM1_ESM.docx]

Table S1. PBA of virus proteins with the HLA alleles used.

| index | allele | HHV1 | HHV2 | HHV3 | HHV4 | HHV5 | HHV6A | HHV6B | HHV7 | HHV8 |
| --- | --- | --- | --- | --- | --- | --- | --- | --- | --- | --- |
| 1 | A*01:01 | 2.813 | 2.120 | 2.659 | 4.605 | 3.507 | 4.605 | 2.659 | 3.219 | 2.659 |
| 2 | A*02:01 | 2.526 | 1.966 | 3.219 | 3.912 | 2.996 | -0.588 | 3.912 | 4.605 | 3.912 |
| 3 | A*02:05 | 2.526 | 2.120 | 2.996 | 3.507 | 3.912 | 0.755 | 3.219 | 3.912 | 3.507 |
| 4 | A*03:01 | 3.507 | 3.219 | 3.219 | 2.526 | 3.912 | 0.968 | 3.219 | 3.912 | 1.833 |
| 5 | A*11:01 | 3.912 | 3.912 | 3.912 | 4.605 | 3.912 | 2.040 | 3.507 | 4.605 | 3.912 |
| 6 | A*23:01 | 1.715 | 1.079 | 2.813 | 2.813 | 3.507 | 2.996 | 3.219 | 3.507 | 3.507 |
| 7 | A*24:02 | 1.966 | 1.309 | 3.507 | 3.507 | 3.507 | 3.507 | 3.507 | 3.507 | 3.507 |
| 8 | A*25:01 | 3.507 | 3.507 | 3.507 | 3.507 | 3.912 | 2.303 | 3.912 | 3.912 | 3.912 |
| 9 | A*26:01 | 2.996 | 2.996 | 3.507 | 3.507 | 3.912 | 1.561 | 3.507 | 3.912 | 3.912 |
| 10 | A*29:01 | 1.514 | 1.833 | 3.912 | 3.219 | 3.912 | 3.507 | 3.219 | 4.605 | 3.912 |
| 11 | A*29:02 | 1.514 | 1.833 | 3.912 | 3.219 | 3.912 | 3.507 | 3.219 | 4.605 | 3.912 |
| 12 | A*30:01 | 3.912 | 3.912 | 4.605 | 3.912 | 3.912 | -0.182 | 3.507 | 2.813 | 2.659 |
| 13 | A*30:02 | 4.605 | 4.605 | 3.912 | 3.912 | 3.912 | 2.659 | 3.219 | 4.605 | 3.219 |
| 14 | A*31:01 | 3.507 | 3.507 | 2.120 | 3.507 | 2.813 | 3.507 | 1.514 | 3.912 | 3.912 |
| 15 | A*32:01 | 3.219 | 3.912 | 3.912 | 3.507 | 4.605 | 2.040 | 3.219 | 3.507 | 3.912 |
| 16 | A*33:01 | 3.912 | 3.912 | 3.507 | 2.408 | 3.912 | 3.507 | 1.897 | 3.912 | 4.605 |
| 17 | A*33:03 | 3.219 | 3.219 | 3.507 | 2.040 | 3.507 | 3.507 | 1.715 | 3.507 | 3.507 |
| 18 | A*36:01 | 3.507 | 3.507 | 2.996 | 2.996 | 3.912 | 4.605 | 3.507 | 3.219 | 2.813 |
| 19 | A*68:01 | 3.507 | 3.507 | 3.507 | 3.507 | 3.912 | 1.966 | 2.659 | 2.813 | 3.507 |
| 20 | A*68:02 | 2.303 | 2.408 | 3.219 | 2.996 | 3.507 | 1.050 | 2.526 | 3.507 | 3.507 |
| 21 | B*07:02 | 1.609 | 1.966 | 3.507 | 3.507 | 2.120 | 2.526 | 3.507 | 2.996 | 2.659 |
| 22 | B*08:01 | 3.219 | 2.996 | 2.408 | 3.912 | 3.219 | 1.661 | 4.605 | 3.219 | 3.219 |
| 23 | B*13:02 | 2.207 | 3.912 | 2.813 | 3.912 | 3.912 | 0.248 | 2.813 | 2.813 | 3.912 |
| 24 | B*14:01 | 3.507 | 3.507 | 2.303 | 3.912 | 3.507 | 3.507 | 2.996 | 3.912 | 3.912 |
| 25 | B*14:02 | 3.507 | 3.507 | 2.303 | 3.912 | 3.507 | 3.507 | 2.996 | 3.912 | 3.912 |
| 26 | B*15:01 | 2.207 | 1.139 | 3.912 | 4.605 | 3.912 | 2.813 | 2.659 | 3.507 | 1.715 |
| 27 | B*15:17 | 2.303 | 3.507 | 3.507 | 2.659 | 3.912 | 2.813 | 3.507 | 3.912 | 3.507 |
| 28 | B*15:18 | 2.408 | 2.408 | 4.605 | 4.605 | 3.219 | 2.996 | 1.966 | 2.996 | 4.605 |
| 29 | B*18:01 | 1.833 | 1.833 | 3.219 | 4.605 | 4.605 | 3.219 | 2.996 | 3.912 | 3.912 |
| 30 | B*27:02 | 3.507 | 1.966 | 3.912 | 2.408 | 2.408 | 2.120 | 2.207 | 2.813 | 3.219 |
| 31 | B*27:05 | 3.219 | 3.507 | 3.912 | 2.040 | 2.996 | 1.833 | 2.120 | 2.996 | 3.507 |
| 32 | B*35:01 | 2.408 | 1.897 | 3.912 | 3.219 | 4.605 | 2.996 | 3.912 | 3.912 | 4.605 |
| 33 | B*35:02 | 3.219 | 2.659 | 2.526 | 3.507 | 2.996 | 3.912 | 4.605 | 3.912 | 3.507 |
| 34 | B*35:03 | 3.507 | 2.526 | 2.408 | 3.507 | 2.526 | 3.912 | 4.605 | 3.912 | 3.507 |
| 35 | B*35:08 | 2.303 | 1.238 | 3.219 | 3.912 | 3.912 | 3.219 | 3.912 | 3.912 | 4.605 |
| 36 | B*37:01 | 2.659 | 2.659 | 2.813 | 3.219 | 3.507 | 2.408 | 3.507 | 3.912 | 3.912 |
| 37 | B*38:01 | 2.526 | 2.996 | 3.912 | 3.912 | 3.912 | 3.507 | 1.171 | 2.120 | 3.507 |
| 38 | B*39:01 | 3.912 | 3.912 | 3.912 | 3.912 | 3.912 | 3.912 | 3.912 | 3.507 | 3.912 |
| 39 | B*39:06 | 3.912 | 3.912 | 3.912 | 3.219 | 3.219 | 4.605 | 3.912 | 2.408 | 3.507 |
| 40 | B*40:01 | 3.219 | 3.219 | 3.912 | 3.507 | 2.996 | 2.040 | 2.813 | 3.219 | 3.912 |
| 41 | B*40:02 | 1.833 | 1.833 | 2.996 | 2.659 | 2.813 | 1.109 | 2.526 | 3.507 | 3.219 |
| 42 | B*41:01 | 2.526 | 1.273 | 2.813 | 3.219 | 2.526 | 0.821 | 2.408 | 2.813 | 3.507 |
| 43 | B*41:02 | 2.996 | 2.996 | 2.207 | 2.659 | 2.996 | 1.109 | 3.507 | 3.507 | 3.912 |
| 44 | B*44:02 | 1.661 | 1.661 | 1.609 | 3.507 | 3.507 | 4.605 | 2.207 | 2.996 | 2.996 |
| 45 | B*44:03 | 1.897 | 1.897 | 1.772 | 3.912 | 3.912 | 4.605 | 1.833 | 3.507 | 3.219 |
| 46 | B*44:05 | 2.659 | 2.659 | 2.526 | 2.659 | 3.912 | 3.912 | 2.813 | 3.912 | 3.507 |
| 47 | B*45:01 | 1.309 | 0.713 | 2.813 | 3.219 | 2.659 | 0.968 | 2.303 | 2.408 | 2.526 |
| 48 | B*47:01 | 2.996 | 2.996 | 3.507 | 3.219 | 3.507 | 3.912 | 2.659 | 4.605 | 4.605 |
| 49 | B*49:01 | 0.994 | 0.994 | 3.507 | 2.659 | 4.605 | 1.661 | 2.040 | 2.996 | 3.912 |
| 50 | B*50:01 | 2.408 | 1.347 | 2.659 | 2.996 | 2.659 | 0.431 | 2.813 | 2.813 | 2.996 |
| 51 | B*51:01 | 3.912 | 3.912 | 3.912 | 2.408 | 3.912 | 3.507 | 2.996 | 3.219 | 3.912 |
| 52 | B*52:01 | 2.813 | 3.507 | 3.912 | 2.303 | 3.507 | 2.303 | 2.813 | 3.507 | 3.912 |
| 53 | B*55:01 | 3.507 | 3.912 | 3.507 | 3.912 | 3.912 | 2.207 | 2.303 | 2.207 | 3.912 |
| 54 | B*56:01 | 3.912 | 3.912 | 3.507 | 3.507 | 3.912 | 2.996 | 2.813 | 2.526 | 3.912 |
| 55 | B*57:01 | 0.916 | 3.507 | 1.897 | 2.303 | 2.207 | 2.813 | 1.661 | 2.996 | 2.207 |
| 56 | B*58:01 | 1.238 | 3.912 | 2.526 | 2.813 | 2.526 | 2.207 | 2.303 | 2.408 | 2.526 |
| 57 | C*01:02 | 3.912 | 3.912 | 2.408 | 3.912 | 3.219 | 2.996 | 3.912 | 2.996 | 3.912 |
| 58 | C*03:03 | 2.120 | 2.303 | 3.912 | 2.813 | 4.605 | 3.912 | 3.507 | 3.219 | 3.507 |
| 59 | C*04:01 | 3.219 | 3.219 | 3.507 | 3.912 | 2.996 | 1.966 | 2.996 | 2.996 | 3.912 |
| 60 | C*05:01 | 2.120 | 2.120 | 2.659 | 4.605 | 3.912 | 2.813 | 2.303 | 3.507 | 2.526 |
| 61 | C*06:02 | 3.507 | 3.219 | 3.219 | 3.219 | 3.219 | 3.219 | 3.912 | 3.912 | 4.605 |
| 62 | C*07:01 | 3.219 | 3.219 | 3.912 | 3.912 | 3.912 | 3.219 | 3.507 | 3.507 | 3.912 |
| 63 | C*07:02 | 3.219 | 3.507 | 3.912 | 3.912 | 4.605 | 3.507 | 4.605 | 4.605 | 4.605 |
| 64 | C*07:04 | 3.912 | 3.507 | 3.507 | 3.912 | 3.912 | 3.219 | 3.507 | 3.912 | 3.912 |
| 65 | C*12:02 | 1.139 | 1.427 | 3.912 | 2.408 | 4.605 | 3.912 | 2.813 | 2.996 | 3.219 |
| 66 | C*12:03 | 2.813 | 3.219 | 3.912 | 2.207 | 4.605 | 3.912 | 2.996 | 3.507 | 3.912 |
| 67 | C*14:02 | 3.912 | 3.912 | 3.219 | 3.912 | 3.912 | 3.507 | 3.912 | 3.912 | 3.912 |
| 68 | C*15:02 | 1.661 | 3.912 | 3.507 | 3.507 | 3.912 | 2.659 | 2.813 | 3.507 | 3.912 |
| 69 | C*16:01 | 2.408 | 2.659 | 3.912 | 2.659 | 4.605 | 3.912 | 3.219 | 2.996 | 3.912 |
| 70 | DPB1*01:01 | 0.139 | 0.635 | 4.605 | 1.609 | 1.386 | -0.262 | 1.079 | 3.912 | 2.120 |
| 71 | DPB1*02:01 | 0.562 | 0.821 | 1.139 | 0.030 | 1.561 | -0.642 | 2.120 | 3.219 | 2.303 |
| 72 | DPB1*02:02 | 0.446 | 0.821 | 1.139 | 0.117 | 1.386 | -0.531 | 2.120 | 2.996 | 2.526 |
| 73 | DPB1*03:01 | -0.693 | -1.459 | 1.514 | 1.309 | 1.897 | -0.182 | 0.400 | -0.262 | 0.916 |
| 74 | DPB1*04:01 | -0.742 | -0.693 | 1.897 | 0.892 | 0.734 | 0.186 | 1.386 | 2.813 | 1.514 |
| 75 | DPB1*04:02 | -0.182 | -0.095 | 1.309 | 0.357 | 0.916 | -0.182 | 1.661 | 3.912 | 1.561 |
| 76 | DPB1*05:01 | 0.094 | -0.182 | 1.347 | 1.609 | 2.996 | -0.182 | 0.734 | 2.659 | 2.120 |
| 77 | DPB1*06:01 | 0.105 | -0.693 | 1.204 | 2.659 | 1.050 | -0.095 | 0.400 | 0.030 | 1.966 |
| 78 | DPB1*09:01 | 0.315 | -0.470 | 2.207 | 2.813 | 0.713 | -0.531 | 0.616 | 0.248 | 2.040 |
| 79 | DPB1*10:01 | 0.186 | -0.693 | 1.309 | 2.526 | 0.892 | -0.182 | 0.580 | 0.186 | 2.120 |
| 80 | DPB1*11:01 | -0.693 | -0.642 | 2.120 | 1.238 | 1.427 | 1.514 | 1.050 | -0.693 | 0.693 |
| 81 | DPB1*13:01 | 0.916 | 0.151 | 1.470 | 2.996 | 0.844 | 0.186 | 2.813 | 0.261 | 0.868 |
| 82 | DPB1*14:01 | -1.065 | -1.526 | 1.715 | 1.204 | 1.661 | -0.182 | 0.777 | 0.186 | 1.204 |
| 83 | DPB1*17:01 | 0.315 | -0.642 | 0.916 | 2.659 | 0.654 | -0.095 | 0.462 | -0.095 | 2.303 |
| 84 | DPB1*19:01 | 0.821 | 0.616 | 1.050 | 0.400 | 1.897 | -1.548 | 1.470 | 4.605 | 0.942 |
| 85 | DQB1*02:01 | 0.916 | 1.833 | 3.912 | 1.273 | 2.659 | 0.198 | 1.386 | -0.182 | 0.211 |
| 86 | DQB1*02:02 | 0.916 | 1.833 | 3.912 | 1.273 | 2.659 | 0.198 | 1.386 | -0.182 | 0.211 |
| 87 | DQB1*03:01 | 1.022 | 1.897 | 0.105 | 1.347 | 0.342 | -1.723 | 0.478 | -1.308 | -0.262 |
| 88 | DQB1*03:02 | -0.405 | 1.022 | 4.605 | 2.659 | 3.912 | 0.892 | 3.912 | 0.288 | 0.030 |
| 89 | DQB1*03:03 | -0.336 | 2.526 | 3.912 | 2.303 | 0.821 | 1.050 | 1.609 | 0.821 | 0.163 |
| 90 | DQB1*04:02 | 1.833 | 2.408 | 2.408 | 2.408 | 0.616 | -0.262 | 2.120 | -0.405 | 1.204 |
| 91 | DQB1*05:01 | 0.301 | -0.470 | 4.605 | 3.219 | 2.040 | 0.868 | 0.799 | 1.966 | -0.182 |
| 92 | DQB1*05:02 | 1.050 | 1.109 | 2.659 | 3.507 | 2.303 | 1.514 | 2.040 | 2.526 | -0.588 |
| 93 | DQB1*05:03 | 2.040 | 1.139 | 2.996 | 4.605 | 1.715 | 2.040 | 1.347 | 2.996 | -0.182 |
| 94 | DQB1*06:01 | -0.470 | -0.531 | 1.347 | 1.309 | 0.868 | -2.398 | 0.892 | -0.788 | 0.994 |
| 95 | DQB1*06:02 | -0.262 | -0.095 | 1.561 | 2.526 | 0.416 | -0.875 | 0.274 | 1.273 | 1.966 |
| 96 | DQB1*06:03 | 0.371 | 1.273 | 0.598 | 2.526 | 1.966 | 2.996 | 0.821 | -0.262 | -0.262 |
| 97 | DQB1*06:04 | -0.336 | -0.470 | 1.273 | 4.605 | 3.507 | 0.248 | 2.408 | -1.281 | -0.405 |
| 98 | DQB1*06:09 | -0.531 | -0.336 | 1.772 | 4.605 | 4.605 | 0.462 | 1.427 | -1.131 | -0.262 |
| 99 | DRB1*01:01 | 0.545 | -0.993 | 0.580 | 1.661 | -0.095 | 1.514 | 1.050 | 0.673 | 2.996 |
| 100 | DRB1*01:02 | 1.204 | -0.262 | -0.095 | 0.211 | 0.545 | -1.099 | 1.897 | 0.151 | 1.715 |
| 101 | DRB1*01:03 | -0.405 | 0.868 | -0.875 | 1.561 | 0.431 | 1.833 | 0.186 | 2.996 | 2.659 |
| 102 | DRB1*03:01 | 0.494 | -0.095 | 2.120 | -0.182 | -0.336 | 0.528 | 0.236 | 0.673 | 0.799 |
| 103 | DRB1*04:01 | -0.095 | 2.813 | 3.507 | 0.580 | 0.545 | 0.342 | 2.040 | 1.347 | -0.405 |
| 104 | DRB1*04:02 | 0.301 | 0.734 | 4.605 | 1.079 | 1.514 | -0.336 | 1.715 | 2.996 | 1.609 |
| 105 | DRB1*04:03 | -0.182 | 0.821 | 4.605 | 0.673 | 0.020 | 0.151 | 0.968 | 2.408 | 0.301 |
| 106 | DRB1*04:04 | -0.182 | 0.261 | 4.605 | 2.040 | 0.713 | 0.799 | 0.163 | 3.507 | 0.062 |
| 107 | DRB1*04:05 | 1.661 | 4.605 | 0.844 | 0.400 | 2.207 | -0.916 | 4.605 | 2.996 | 2.996 |
| 108 | DRB1*04:07 | -0.182 | 2.526 | 2.813 | 0.844 | 1.204 | 0.673 | 3.507 | 1.514 | 1.427 |
| 109 | DRB1*04:08 | -0.262 | 2.408 | 3.507 | 0.713 | 1.897 | 0.994 | 3.507 | 2.207 | 0.248 |
| 110 | DRB1*07:01 | -0.588 | -0.405 | 0.734 | 2.526 | 1.772 | -0.642 | 0.844 | 3.912 | 1.897 |
| 111 | DRB1*08:01 | 3.219 | 3.219 | 1.386 | 1.139 | 0.968 | 1.139 | 4.605 | 2.120 | 1.238 |
| 112 | DRB1*08:03 | 3.219 | 3.219 | 0.478 | 0.635 | 0.654 | 1.609 | 4.605 | 3.219 | 2.040 |
| 113 | DRB1*09:01 | 1.022 | 1.309 | 1.772 | 1.171 | 1.833 | -0.588 | 0.994 | 4.605 | 2.813 |
| 114 | DRB1*10:01 | 1.079 | 1.609 | 0.892 | 0.580 | 0.968 | 0.163 | 0.117 | 2.408 | 0.968 |
| 115 | DRB1*11:01 | 3.507 | 2.659 | 4.605 | 0.462 | 0.580 | 4.605 | 0.371 | 1.386 | 1.386 |
| 116 | DRB1*11:02 | 0.580 | 2.659 | 0.274 | 0.051 | 1.715 | -0.956 | -0.693 | 2.526 | 1.204 |
| 117 | DRB1*11:03 | 0.462 | 1.833 | 0.693 | 0.274 | 0.734 | -1.308 | -0.833 | 0.693 | 3.219 |
| 118 | DRB1*11:04 | 0.041 | -0.470 | 1.109 | 0.528 | 1.386 | -0.531 | -0.095 | 0.799 | 2.040 |
| 119 | DRB1*12:01 | -0.182 | 0.051 | 4.605 | -0.531 | 0.942 | -0.182 | 3.912 | 0.693 | 1.470 |
| 120 | DRB1*13:01 | 0.580 | 2.659 | 0.274 | 0.051 | 1.715 | -0.956 | -0.693 | 2.526 | 1.204 |
| 121 | DRB1*13:02 | 0.446 | 1.514 | 1.514 | 0.821 | 2.408 | 1.514 | 0.616 | 2.207 | 1.204 |
| 122 | DRB1*13:03 | 1.609 | 1.897 | 0.673 | 3.507 | 1.238 | 1.966 | 3.507 | 1.079 | 0.151 |
| 123 | DRB1*13:05 | 3.507 | 2.659 | 4.605 | 0.462 | 0.580 | 4.605 | 0.371 | 1.386 | 1.386 |
| 124 | DRB1*14:01 | -0.470 | -0.336 | 0.431 | 4.605 | 2.408 | 0.635 | -0.470 | 2.813 | 0.892 |
| 125 | DRB1*15:01 | 1.386 | 0.511 | 3.912 | 3.507 | 3.219 | 3.507 | 2.408 | 4.605 | 0.117 |
| 126 | DRB1*15:02 | 3.912 | 3.507 | 1.661 | 3.219 | 4.605 | 2.526 | 2.996 | 2.996 | 0.400 |
| 127 | DRB1*16:01 | 0.416 | 0.236 | 0.446 | 2.207 | 2.207 | 1.715 | 1.897 | 2.207 | 1.715 |

Table S2. Amino acid sequences of the 9 HHV proteins used. Labels in headers are from the Uniprot database (https://www.uniprot.org/)

| HHV-1: Q69091 · GD_HHV11 | Envelope glycoprotein D | 394 AA |
| --- | --- | --- |

MGGAAARLGAVILFVVIVGLHGVRSKYALVDASLKMADPNRFRGKDLPVLDQLTDPPGVRRVYHIQAGLPDPFQPPSLPITVYYAVLERACRSVLLNAPSEAPQIVRGASEDVRKQPYNLTIAWFRMGGNCAIPITVMEYTECSYNKSLGACPIRTQPRWNYYDSFSAVSEDNLGFLMHAPAFETAGTYLRLVKINDWTEITQFILEHRAKGSCKYALPLRIPPSACLSPQAYQQGVTVDSIGMLPRFIPENQRTVAVYSLKIAGWHGPKAPYTSTLLPPELSETPNATQPELAPEDPEDSALLEDPVGTVAPQIPPNWHIPSIQDAATPYHPPATPNNMGLIAGAVGGSLLAALVICGIVYWMRRHTQKAPKRIRLPHIREDDQPSSHQPLFY

| HHV-2: P03172 · GD_HHV23 | Envelope glycoprotein D | 393 AA |
| --- | --- | --- |

MGRLTSGVGTAALLVVAVGLRVVCAKYALADPSLKMADPNRFRGKNLPVLDRLTDPPGVKRVYHIQPSLEDPFQPPSIPITVYYAVLERACRSVLLHAPSEAPQIVRGASDEARKHTYNLTIAWYRMGDNCAIPITVMEYTECPYNKSLGVCPIRTQPRWSYYDSFSAVSEDNLGFLMHAPAFETAGTYLRLVKINDWTEITQFILEHRARASCKYALPLRIPPAACLTSKAYQQGVTVDSIGMLPRFIPENQRTVALYSLKIAGWHGPKPPYTSTLLPPELSDTTNATQPELVPEDPEDSALLEDPAGTVSSQIPPNWHIPSIQDVAPHHAPAAPSNPGLIIGALAGSTLAVLVIGGIAFWVRRRAQMAPKRLRLPHIRDDDAPPSHQPLFY

| HHV-3: Q9J3M8 · GE_VZVO | Envelope glycoprotein E | 623 AA |
| --- | --- | --- |

MGTVNKPVVGVLMGFGIITGTLRITNPVRASVLRYDDFHIDEDKLDTNSVYEPYYHSDHAESSWVNRGESSRKAYDHNSPYIWPRNDYDGFLENAHEHHGVYNQGRGIDSGERLMQPTQMSAQEDLGDDTGIHVIPTLNGDDRHKIVNVDQRQYGDVFKGDLNPKPQGQRLIEVSVEENHPFTLRAPIQRIYGVRYTETWSFLPSLTCTGDAAPAIQHICLKHTTCFQDVVVDVDCAENTKEDQLAEISYRFQGKKEADQPWIVVNTSTLFDELELDPPEIEPGVLKVLRTEKQYLGVYIWNMRGSDGTSTYATFLVTWKGDEKTRNPTPAVTPQPRGAEFHMWNYHSHVFSVGDTFSLAMHLQYKIHEAPFDLLLEWLYVPIDPTCQPMRLYSTCLYHPNAPQCLSHMNSGCTFTSPHLAQRVASTVYQNCEHADNYTAYCLGISHMEPSFGLILHDGGTTLKFVDTPESLSGLYVFVVYFNGHVEAVAYTVVSTVDHFVNAIEERGFPPTAGQPPATTKPKEITPVNPGTSPLLRYAAWTGGLAAVVLLCLVIFLICTAKRMRVKAYRVDKSPYNQSMYYAGLPVDDFEDSESTDTEEEFGNAIGGSHGGSSYTVYIDKTR

| HHV-4: P03188 · GB_EBVB9 | Envelope glycoprotein B | 857 AA |
| --- | --- | --- |

MTRRRVLSVVVLLAALACRLGAQTPEQPAPPATTVQPTATRQQTSFPFRVCELSSHGDLFRFSSDIQCPSFGTRENHTEGLLMVFKDNIIPYSFKVRSYTKIVTNILIYNGWYADSVTNRHEEKFSVDSYETDQMDTIYQCYNAVKMTKDGLTRVYVDRDGVNITVNLKPTGGLANGVRRYASQTELYDAPGWLIWTYRTRTTVNCLITDMMAKSNSPFDFFVTTTGQTVEMSPFYDGKNKETFHERADSFHVRTNYKIVDYDNRGTNPQGERRAFLDKGTYTLSWKLENRTAYCPLQHWQTFDSTIATETGKSIHFVTDEGTSSFVTNTTVGIELPDAFKCIEEQVNKTMHEKYEAVQDRYTKGQEAITYFITSGGLLLAWLPLTPRSLATVKNLTELTTPTSSPPSSPSPPAPSAARGSTPAAVLRRRRRDAGNATTPVPPTAPGKSLGTLNNPATVQIQFAYDSLRRQINRMLGDLARAWCLEQKRQNMVLRELTKINPTTVMSSIYGKAVAAKRLGDVISVSQCVPVNQATVTLRKSMRVPGSETMCYSRPLVSFSFINDTKTYEGQLGTDNEIFLTKKMTEVCQATSQYYFQSGNEIHVYNDYHHFKTIELDGIATLQTFISLNTSLIENIDFASLELYSRDEQRASNVFDLEGIFREYNFQAQNIAGLRKDLDNAVSNGRNQFVDGLGELMDSLGSVGQSITNLVSTVGGLFSSLVSGFISFFKNPFGGMLILVLVAGVVILVISLTRRTRQMSQQPVQMLYPGIDELAQQHASGEGPGINPISKTELQAIMLALHEQNQEQKRAAQRAAGPSVASRALQAARDRFPGLRRRRYHDPETAAALLGEAETEF

| HHV-5: P06473 · GB_HCMVA | Envelope glycoprotein B | gB | 906 AA |
| --- | --- | --- | --- |

MESRIWCLVVCVNLCIVCLGAAVSSSSTSHATSSTHNGSHTSRTTSAQTRSVYSQHVTSSEAVSHRANETIYNTTLKYGDVVGVNTTKYPYRVCSMAQGTDLIRFERNIICTSMKPINEDLDEGIMVVYKRNIVAHTFKVRVYQKVLTFRRSYAYIYTTYLLGSNTEYVAPPMWEIHHINKFAQCYSSYSRVIGGTVFVAYHRDSYENKTMQLIPDDYSNTHSTRYVTVKDQWHSRGSTWLYRETCNLNCMLTITTARSKYPYHFFATSTGDVVYISPFYNGTNRNASYFGENADKFFIFPNYTIVSDFGRPNAAPETHRLVAFLERADSVISWDIQDEKNVTCQLTFWEASERTIRSEAEDSYHFSSAKMTATFLSKKQEVNMSDSALDCVRDEAINKLQQIFNTSYNQTYEKYGNVSVFETSGGLVVFWQGIKQKSLVELERLANRSSLNITHRTRRSTSDNNTTHLSSMESVHNLVYAQLQFTYDTLRGYINRALAQIAEAWCVDQRRTLEVFKELSKINPSAILSAIYNKPIAARFMGDVLGLASCVTINQTSVKVLRDMNVKESPGRCYSRPVVIFNFANSSYVQYGQLGEDNEILLGNHRTEECQLPSLKIFIAGNSAYEYVDYLFKRMIDLSSISTVDSMIALDIDPLENTDFRVLELYSQKELRSSNVFDLEEIMREFNSYKQRVKYVEDKVVDPLPPYLKGLDDLMSGLGAAGKAVGVAIGAVGGAVASVVEGVATFLKNPFGAFTIILVAIAVVIITYLIYTRQRRLCTQPLQNLFPYLVSADGTTVTSGSTKDTSLQAPPSYEESVYNSGRKGPGPPSSDASTAAPPYTNEQAYQMLLALARLDAEQRAQQNGTDSLDGQTGTQDKGQKPNLLDRLRHRKNGYRHLKDSDEEENV

| HHV-6A: P0DOE0 · GQ2_HHV6U | Envelope glycoprotein Q2 | 214 AA |
| --- | --- | --- |

MHFLVVYILIHFHAYRGMAALPLFSTLPKITSCCDSYVVINSSTSVSSLISTCLDGEILFQNEGQKFCRPLTDNRTIVYTMQDQVQKPLSVTWMDFNLVISDYGRDVINNLTKSAMLARKNGPRYLQMENGPRYLQMETRISDLFRHECYQDNYYVLDKKLQMFYPTTHSNELLFYPSEATLPSPWQEPPFSSPWPEPTFPSRWYWLLLNYTNY

| HHV-6B: Q9QJ11 · GQ1_HHV6Z | Envelope glycoprotein Q1 | 516 AA |
| --- | --- | --- |

MRPPRRSAPILVCAISMATALSNATVHRDAGTVESTPPPDDEDNYTAKYYDDSIYFNIYDGTNPTPRRRTLPEIISKFSTSEMSRLGGLKAFVPVDYTPTTTLEDIEDLLNYAICDDNSCGCLIETEARXMFGDIIICVPLSAESRGVRNLKSRIMPMGLSQILSSGLGLHFSLLYGAFGSNYNSLAYMERLKPLTAMTAIAFCPMTSKLELRQNYRLEKARXNLIVNIELLKIQNHGGQTIKTLTSFAIVRKDSDGQDWETCTRFASVSIEDILRSKPAANGTCCPPRDVHHDRPTLQSSNSWTRTEYFEPWQDVVDAYVPINDNHCPNDSYVVFQTLQGHEWCSRLNKNDTKNYLSSVLAFKNALYETEELMETIGMRLASQILSLVGQRGTSIRNIDPAIVSALWHSLPEKLTTTNIKYDIASPTHMSPALXTIFIQTGTSKQRFRNAGLLMVNNIFTVQARYSKQNMFEKKIYGYEHLGQALCEGGHVFYNPRDVYFQNIKMAATEPTVVRT

| HHV-7: P52353 · GH_HHV7J | Envelope glycoprotein H | 690 AA |
| --- | --- | --- |

MYFYINSLLLIVSINGWKHWNILNSSICVNEKTNQTIIQPGLITFNFHDYNETRVYQIPKCLFGYTFVSNLFDSVNFDESFDQYKHRITRFFNPSTEKAVKIYAQKFQTNIKPVSHTKTITVSFLPLFYEKDVYFANVSEIRKLYYNQYICTLSNGLTDYLFPITERCVMRHYNYLNTVFMLALTPSFFIISVETGMDDVVFIFGNVSRIFFKAPFRKSSFIYRQTVSDDLLLITKKTTIERFYPFLKIDFLDDIWKQNYDISFLIAKFNKLATVYIMEGFCGKPVNKDTFHLMFLFGLTHFLYSTRGDGLLPLLEILNTHQSIITMGRFLEKCFKMTKSHLLYPEMEKLQNFQLVDYSYITSDLTIPISAKLAFLSLADGRIVTVPQNKWKEIENNIETLYEKHKLFTNLTQPERANLFLLSEIGNSLVFQEKIKRKIHVLLASLCNPLEMYFWTHMLDNVMDIETMFSPCATATRKDLTQRVVNNILSYKNLDAYTNKVMNTLSVYRKKRLDMFKSISCVSNEQAAFLTLPNITYTISSKYILAGTSFSVTSTVISTTIIITVVPLNSTCTPTNYKYSVKNIKPIYNISSHDCVFCESLVVEYDDIDGIIQFVYIMDDKQLLKLIDPDTNFIDVNPRTHYLLFLRNGSVFEITALDLKSSQVSIMLVLLYLIIIIIVLFGIYHVFRLF

| HHV-8: F5HAK9 · GH_HHV8P | Envelope glycoprotein H | 730 AA |
| --- | --- | --- |

MQGLAFLAALACWRCISLTCGATGALPTTATTITRSATQLINGRTNLSIELEFNGTSFFLNWQNLLNVITEPALTELWTSAEVAEDLRVTLKKRQSLFFPNKTVVISGDGHRYTCEVPTSSQTYNITKGFNYSALPGHLGGFGINARLVLGDIFASKWSLFARDTPEYRVFYPMNVMAVKFSISIGNNESGVALYGVVSEDFVVVTLHNRSKEANETASHLLFGLPDSLPSLKGHATYDELTFARNAKYALVAILPKDSYQTLLTENYTRIFLNMTESTPLEFTRTIQTRIVSIEARRACAAQEAAPDIFLVLFQMLVAHFLVARGIAEHRFVEVDCVCRQYAELYFLRRISRLCMPTFTTVGYNHTTLGAVAATQIARVSATKLASLPRSSQETVLAMVQLGARDGAVPSSILEGIAMVVEHMYTAYTYVYTLGDTERKLMLDIHTVLTDSCPPKDSGVSEKLLRTYLMFTSMCTNIELGEMIARFSKPDSLNIYRAFSPCFLGLRYDLHPAKLRAEAPQSSALTRTAVARGTSGFAELLHALHLDSLNLIPAINCSKITADKIIATVPLPHVTYIISSEALSNAVVYEVSEIFLKSAMFISAIKPDCSGFNFSQIDRHIPIVYNISTPRRGCPLCDSVIMSYDESDGLQSLMYVTNERVQTNLFLDKSPFFDNNNLHIHYLWLRDNGTVVEIRGMYRRRAASALFLILSFIGFSGVIYFLYRLFSILY
